# High‐resolution mapping of the pericentromeric region on wheat chromosome arm 5AS harbouring the Fusarium head blight resistance QTL *Qfhs.ifa‐5A*

**DOI:** 10.1111/pbi.12850

**Published:** 2017-11-10

**Authors:** Maria Buerstmayr, Barbara Steiner, Christian Wagner, Petra Schwarz, Klaus Brugger, Delfina Barabaschi, Andrea Volante, Giampiero Valè, Luigi Cattivelli, Hermann Buerstmayr

**Affiliations:** ^1^ Department of Agrobiotechnology Tulln BOKU ‐ University of Natural Resources and Life Sciences, Vienna Tulln Austria; ^2^ Council for Agricultural Research and Economics (CREA) Genomics Research Centre Fiorenzuola d'Arda Italy; ^3^ Council for Agricultural Research and Economics (CREA) Research Centre for Cereal and Industrial Crops Vercelli Italy

**Keywords:** radiation selfing, radiation hybrid mapping, Fusarium head blight, *Triticum aestivum*, suppressed recombination, gamma irradiation

## Abstract

The *Qfhs.ifa‐5A* allele, contributing to enhanced Fusarium head blight resistance in wheat, resides in a low‐recombinogenic region of chromosome 5A close to the centromere. A near‐isogenic RIL population segregating for the *Qfhs.ifa‐5A* resistance allele was developed and among 3650 lines as few as four recombined within the pericentromeric C‐5AS1‐0.40 bin, yielding only a single recombination point. Genetic mapping of the pericentromeric region using a recombination‐dependent approach was thus not successful. To facilitate fine‐mapping the physically large *Qfhs.ifa‐5A* interval, two gamma‐irradiated deletion panels were generated: (i) seeds of line NIL3 carrying the *Qfhs.ifa‐5A* resistance allele in an otherwise susceptible background were irradiated and plants thereof were selfed to obtain deletions in homozygous state and (ii) a radiation hybrid panel was produced using irradiated pollen of the wheat line Chinese Spring (CS) for pollinating the CS‐nullisomic5Atetrasomic5B. In total, 5157 radiation selfing and 276 radiation hybrid plants were screened for deletions on 5AS and plants containing deletions were analysed using 102 5AS‐specific markers. Combining genotypic information of both panels yielded an 817‐fold map improvement (cR/cM) for the centromeric bin and was 389‐fold increased across the *Qfhs.ifa‐5A* interval compared to the genetic map, with an average map resolution of 0.77 Mb/cR. We successfully proved that the RH mapping technique can effectively resolve marker order in low‐recombining regions, including pericentromeric intervals, and simultaneously allow developing an *in vivo* panel of sister lines differing for induced deletions across the *Qfhs.ifa‐5A* interval that can be used for phenotyping.

## Introduction

The allohexaploid wheat *Triticum aestivum* L. (2*n* = 6*x* = 42) consists of three homoeologous subgenomes (A, B and D) of seven chromosomes each (Petersen *et al*., 2006) and has an estimated genome size of ~17 Gb, about 40 times the size of the rice genome (Bennett and Smith, 1976; Doležel *et al*., 2009). More than 80% of the nuclear DNA consists of highly repetitive transposable elements (TEs), and the protein‐coding regions account for 2%–3% only (Paux *et al*., 2006). These particular features substantially compromise molecular genetic methods for genome assembly, map construction, fine‐mapping and positional cloning. Most commonly, genetic linkage maps have been developed to determine position of markers and associated traits; marker orders and distances are deduced from the frequency of recombination between markers during crossover of homologous chromosomes. Crossover events are distributed unevenly along the chromosome. Less than 1% of the recombination occurs in 25%–40% of the chromosomal regions around the centromere; the recombination frequency increases exponentially with distance from the centromere (Akhunov *et al*., 2003; Erayman *et al*., 2004; Saintenac *et al*., 2009). Similarly, distribution of genes along the chromosomes is uneven with higher densities observed in the distal regions (Erayman *et al*., 2004; Linkiewicz *et al*., 2004; Pingault *et al*., 2015). Studying gene content, gene density and distribution, it was estimated that more than 30% of the wheat genes are in recombination‐poor regions (Erayman *et al*., 2004). As a consequence, high‐resolution mapping and positional cloning of these genes employing recombination‐dependent linkage is practically impossible. Even highly sophisticated mapping approaches such as population sequencing (POPSEQ) that are able to generate millions of markers (Chapman *et al*., 2015) depend on meiotic recombination and are thus confronted with the same limitations. To overcome these constraints, an alternative, recombination‐independent mapping approach is required.

Radiation hybrid (RH) mapping utilizes ionizing radiation to generate double‐strand breaks (DSB), which are among the most severe type of DNA damages, and that if unrepaired lead to the loss of the entire segment distal of the break causing terminal deletions. Plants have developed different DNA repair pathways to maintain genomic stability (Manova and Gruszka, 2015; Yoshiyama *et al*., 2013). The nonhomologous end‐joining (NHEJ) is an efficient way to repair DSBs and re‐joins breaks in a sequence‐independent manner (Knoll *et al*., 2014; Pipiras *et al*., 1998; Sargent *et al*., 1997). This repair pathway is error prone and can cause various kinds of genomic rearrangements such as interstitial deletions, insertions, inversions and translocations (Pipiras *et al*., 1998; Puchta, 2005). Ionizing radiation‐induced chromosomal breaks occur randomly and are evenly distributed across the entire chromosomes, including pericentromeric regions (Kumar *et al*., 2012; Tiwari *et al*., 2016). Data matrix consists of presence and absence information of polymorphism‐independent specific sequences, allowing the utilization of any type of marker. RH mapping played a major role in whole‐genome sequencing and assembly of human (Lander *et al*. 2001) and animal genomes (Faraut *et al*., 2009). Moreover, RH mapping has successfully supported high‐resolution mapping of individual wheat chromosomes 1D (Kalavacharla *et al*., 2006), 3B (Kumar *et al*., 2012; Paux *et al*., 2008), 6B (Kobayashi *et al*., 2015) and 4A (Balcárková *et al*., 2017), the D‐subgenome (Kumar *et al*. 2015 Riera‐Lizarazu *et al*., 2010) and the whole genome of the hexaploid wheat (Tiwari *et al*., 2016).

The allohexaploid genome of wheat is capable to tolerate large chromosomal aberrations (Endo and Gill, 1996; Sears, 1966; Sears and Sears, 1978), allowing the development of viable and genetically stable lines despite lacking large chromosomal segments. RH mapping takes advantage of this plasticity; viable mutant lines are amenable for forward and reverse genetic studies and provide an important source for fine‐mapping and positional cloning.

*Qfhs.ifa‐5A* (Buerstmayr *et al*., 2003) and *Qfhi.nau*‐5A, syn *Fhb5* (Lin *et al*., 2006; Xue *et al*., 2011) are major resistance QTL for Fusarium head blight (FHB) and both mapped close to the centromere of chromosome 5A. The importance of this genomic interval for the presence of gene(s) affecting FHB resistance was supported by the identification of colocating resistance QTL derived from at least nine independent resistance donors (Buerstmayr *et al*., 2009). Xue *et al*. (2011) fine‐mapped *Fhb5* to the short arm of the chromosome 5A spanning an interval that covered 75% of the physical length of chromosome 5AS. A substantially improved map resolution of this long chromosomal segment is essential for precisely locating the QTL. While genetic mapping is an excellent tool for identifying QTL, it is, for obvious reasons, less suitable to fine‐map low‐recombining regions.

On that account, we supplemented recombination‐based fine‐mapping of *Qfhs.ifa‐5A* using near‐isogenic recombinant inbred lines (NI‐RILs) with RH mapping technique. For this, two sets of gamma‐irradiated wheat panels were developed, differing in both plant source and irradiation approach applied. One panel originated from irradiated seeds of the line NIL3 carrying the *Qfhs.ifa‐5A* resistance allele in the background of the susceptible cultivar Remus and was intended to generate a series of sister lines of randomly induced deletions across the *Qfhs.ifa‐5A* interval. Beyond fine‐mapping, selected mutant lines obtained from the NIL3 panel will be used for phenotyping with the aim to precisely locate *Qfhs.ifa‐5A*. Fine‐mapping of the QTL interval was further enhanced with an RH panel generated by pollinating CS‐nullisomic5Atetrasomic5B (CS‐N5AT5B) (Sears, 1966) plants with γ‐irradiated CS pollen. Mapping results with the NI‐RIL population and of the two panels were compared. Finally, the RH map was compared to the recently published 5A consensus genetic map (called ‘neighbour map’) (Barabaschi *et al*., 2015).

## Results

### Deletion bin mapping of chromosome 5A

A total of 169 markers were screened for their physical position on chromosome 5A. Detailed information on the markers tested, their assignment to 5AL or 5AS and, within 5AS, to a specific physical deletion bin, polymorphism between Remus and CM‐82036 and type of marker is listed in Table S1. Among the tested markers, 118 mapped to the short arm and 17 to the long arm; 34 did not map to 5A. Twenty‐four markers (17 on 5AS and 7 on 5AL) were polymorphic between Remus and CM‐82036. Of the markers assigned to the short arm 52, 34 and 32 mapped to the bin intervals C‐5AS1‐0.40, 5AS1‐0.40‐0.75 and 5AS3‐0.75‐0.97, respectively. Insertion site‐based polymorphism (ISBP), simple sequence repeat (SSR) and conserved ortholog set (COS) markers mapped across all bins, repeat junction markers (RJMs) mapped to bins C‐5AS1‐0.40, 5AS1‐0.40‐0.75 and single nucleotide polymorphism markers (SNPs) mapped to bins 5AS1‐0.40‐0.75, 5AS3‐0.75‐0.97.

### An updated genetic map for the *Qfhs.ifa‐5A* locus using DH and NI‐RIL populations

**Figure 1:**
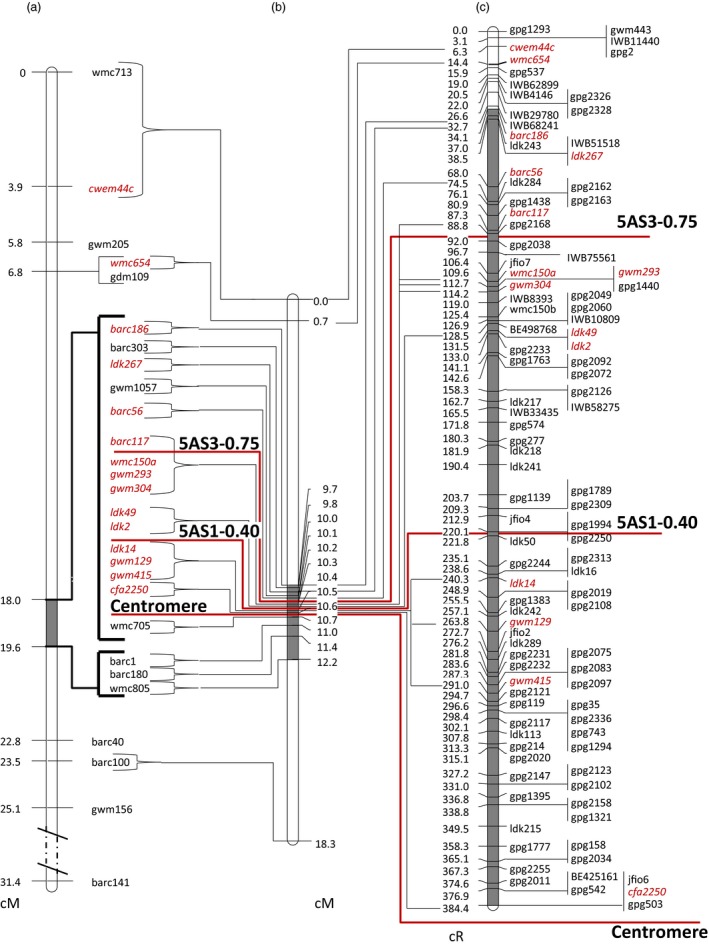
Maps of the wheat chromosome 5AS. (a) Genetic map of the refined linkage group of the DH population (Buerstmayr *et al*., 2003); (b) genetic map of NI‐RIL population; (c) RSH‐consensus map. Markers included in all three maps are in *red*. Position of centromere and proximal borders of physical deletion bins are indicated by red solid lines. Highlighted grey intervals in chromosome bars refer to the *Qfhs.ifa‐5A* interval.

To increase the density of markers at the *Qfhs.ifa‐5A* interval, thirteen markers (ldk2, ldk14, ldk49, ldk267, barc303, cfa2250, cwem44c, gwm415, wmc150, wmc654, wmc705, wmc713 and wmc805) selected as being polymorphic were added to the existing 5A linkage group of the Remus × CM‐82036 DH population (Buerstmayr *et al*., 2003). The enhanced new genetic map contained 28 markers, comprising 15 previously mapped and 13 newly added markers (Figure S1, Figure 1a). However, the added markers did not increase map resolution as all of them cosegregated with one of the markers already positioned in the *Qfhs.ifa‐5A* interval. The refined linkage group yielded 10 recombination points covering a genetic distance of 31.4 cM. Marker order was in agreement with their physical bin allocation. Three groups of cosegregant markers were identified located on the short arm (two markers), in the centromeric region (16 markers) and on the long arm (three markers). The centromeric cluster included markers which are either assigned to deletion bins 5AS3‐0.75‐0.97, 5AS1‐0.40‐0.75, C‐5AS1‐0.40 on the short arm or 5AL5‐0.46‐0.55 on the long arm, and accordingly refer to >70% of the physical length of the 5A chromosome. All analysed *Fusarium* resistance traits coincided and mapped within a 1.6‐cM QTL support interval flanked by barc186 (assigned to 5AS3‐0.75‐0.97) and wmc805 (assigned to 5AL5‐0.46‐0.55) (Figure S1; Table S2); thus, we can conclude that this level of resolution was largely unsatisfactory to dissect the *Qfhs.ifa‐5A* locus.

Attempts at improving the resolution around *Qfhs.ifa‐5A* were further pursued through screening 3650 F_2_ plants of a NI‐RIL population for recombination between the QTL flanking markers. A total of 70 plants recombining within the target interval were identified and genotyped using the identified polymorphic markers. Markers partitioned the linkage group into 15 subintervals for a total length of 18.3 cM (10.6 cM on 5AS, 7.7 cM on 5AL) (Figure 1b, Table S3.1). The QTL, with barc186 and wmc805 as flanking markers, spanned across the centromere and covered a distance of 2.5 cM. Of the 15 subintervals, 12 were located within the QTL interval. The cluster of 16 markers cosegregating in the DH population was partitioned into nine subintervals. Even in the higher‐resolution map, three marker clusters remained unresolved: barc117, wmc150a, gwm304 and gwm293, ldk2 and ldk49 as well as gwm129, gwm415 and ldk14 (Figure 1b). Of 3650 NI‐RILs screened, only 4, 12 and 14 lines recombined within the centromeric, interstitial and distal bin, respectively, using barc186 as flanking marker (Table S3.1).

### Radiation hybrid mapping of the short arm of chromosome 5A using two different panels (RS‐NIL3 and RH‐CS)

All members of the panels RS‐NIL3 (5157 genotypes) and RH‐CS (276 genotypes) were initially surveyed using 15 and 35 5AS‐specific markers, respectively. On average, 1.84% of all analysed RS‐NIL3 plants showed detectable deletions. Proportion of plants containing deletions was homogeneous (chi‐square test, *P *=* *0.57) and ranged from 1.04% to 2.38% among the 240, 250, 270, 300, 330 and 350 Gy plants (Table S4). The percentage was significantly higher in the pollen‐irradiated RH‐CS panel with 18.12% containing deletions. Based on prescreening results, all identified genotypes having ≥1 5AS‐specific markers deleted were selected and genotyped using a set of 102 markers assigned to 5AS composed of 57 ISBP, 28 SSR, 11 SNP, 4 RJM and 2 COS markers and three SSR markers assigned to 5AL.

Fine‐mapping of the RS‐NIL3 subpanel confirmed the presence of deletions in all selected 95 plants. Fifteen pairs of plants, each descending from a single M_1_ seed, had the same markers deleted and thus likely represented duplicates of the same deletion event and were therefore merged to represent a single genotype. Two plants had all 5AS markers deleted, while markers on the long arm were retained, indicating that deletions did not stretch across the centromere.

Genotyping the RH‐CS subpanel verified marker losses in all selected 50 plants. Ten of the plants lost all markers (including those on 5AL) and thus most likely the entire 5A chromosome; these plants were therefore excluded from further analysis. Excluding duplicate genotypes and genotypes having either none or all markers retained, a total of 80 and 40 genotypes for the RS‐NIL3 and RH‐CS panels, respectively, were considered as informative and used for subsequent analysis and statistics calculations.

**Figure 2:**
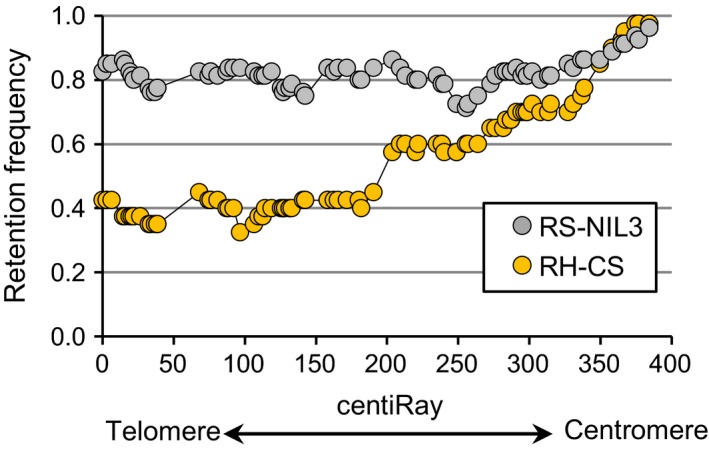
Retention frequencies at each marker along the 5AS chromosome of RS‐NIL3 and RH‐CS panel.

**Figure 3:**
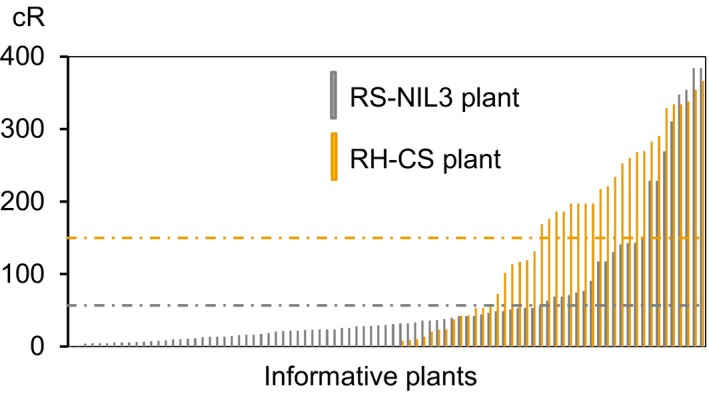
Deletion lengths of informative plants of RS‐NIL3 and RH‐CS panel. Mean deletion lengths are indicated by a dashed grey line (RS‐NIL 3 panel) and a dashed yellow line (RH‐CS panel).

The calculated overall retention rate across datasets was 0.74 (Table 1), with RS‐NIL3 panel having on average 26% more markers retained (retention frequency 0.82) than the RH‐CS panel (retention frequency 0.56). The retention frequency for individual markers ranged from 0.71 to 0.96 in the RS‐NIL3 panel and from 0.33 to 0.97 in the RH‐CS panel. Along the chromosome, the retention frequency differed markedly between the RS‐NIL3 and the RH‐CS panel (Figure 2). There was a steady increase in markers loss from proximal to distal in the RH‐CS panel, unlike in the RS‐NIL3 panel, where marker loss varied only slightly along the chromosome. In both panels, the retention frequency was highest for the markers closest to the centromere (Figure 2). Apart from seven plants with two and one plant with three separate aberrations, all remaining plants had only one deleted chromosomal segment (Table S3.2). Notably, while in the RS‐NIL3 panel 84% of the deletions were of interstitial type, terminal deletions (55%) dominated in the RH‐CS panel. Two chromosomal breaks are required for interstitial deletions, while terminal deletions result from a single break followed by the loss of the entire fragment distal to the break. Consequently, relatively more obligate breaks were found in the RS‐NIL3 (160) than in the RH‐CS panels (61). The map of RS‐NIL3 panel was longer than the map of RH‐CS panel (541.4 cR versus 272 cR), and the consensus map calculated a total length of 384.4 cR. A remarkable difference in sizes of deleted fragments was observed between the two panels, with a mean deletion length being 2.6 times higher in RH‐CS (164.4 cR) than in RS‐NIL3 panel (63.5 cR) (Table 1; Figure 3). Additional information regarding number and range of breakpoints per marker and line, average distances and ranges between loci, length and ranges of deleted fragments are summarized in Table 1.

**Table 1:** Summary statistic of RS‐NIL3, RH‐CS and RSH‐consensus map based on genotyping informative lines with 102 markers

Panel	RS‐NIL3	RH‐CS	RSH
	Mean (range)	Mean (range)	Mean (range)
No. informative lines	80	40	120
Avg. retention frequency	0.82	0.56	0.74
Avg. retention frequency/line	0.82 (0–0.98)	0.56 (0.05–0.98)	0.74 (0.0–0.98)
Avg. retention frequency/marker	0.82 (0.71–0.96)	0.56 (0.33–0.97)	0.74 (0.63–0.97)
No. markers	102	102	102
No. double markers	35	63	26
No. mapped loci	67	39	76
Map size (cR)	541.4	272	384.4
Markers per locus	1.52 (1–4)	2.62 (1–9)	1.34 (1–4)
Distances between loci (cR)	8.2 (2.7–50.8)	7.2 (3.9–24.6)	5.13 (1.4–29.5)
No. terminal deletion	14	23	37
No. interstitial deletion	73	19	92
No. breakpoints	160	61	221
Breakpoints/line	2 (1–6)	1.52 (1–4)	1.84 (1–6)
Breakpoints/loci	2.39 (1–12)	1.56 (1–5)	2.91 (1–16)
Mean deletion length a(cR)	63.5 (1.6–384.4)	164.4 (7.8–366.2)	95.7 (1.6–384.4)

a mean lengths of deletions are calculated as the average of maximum length (distance between the position of retained markers flanking the deletions) and minimum length (distance between the position of deleted markers flanking the deletions) based on cR distances of the RSH‐consensus map.

Considering the 384.4 cR (obtained for the RSH‐consensus map) and the 295 Mb as the size of the 5AS chromosome (Paux *et al*., 2008), and assuming a uniform distribution of obligate breaks, then 1 cR corresponds to ~0.77 Mb. Using the calculated 0.77 Mb/cR and a mean distance of 5.13 cR between loci, an average physical distance of ~3.95 Mb for neighbouring loci was estimated. Map resolution among the centromeric, the interstitial and the distal bin varied only slightly (0.72, 0.82 and 0.69 Mb/cR, respectively) (Table 2), thus indicating a homogeneous map resolution across the linkage group and suggesting that cR are meaningful predictors for Mb distances.

**Table 2:** Estimates of Mb, cR and cM and ratio of Mb to cR, Mb to cM and cR to cM, given for each physical bin separately and across the short arm of 5A

Physical deletion bin	Mb^a^	cR^b^	cM^c^	Mb/cR	Mb/cM	cR/cM
Centromeric bin C‐5AS1‐0.40	118.00	163.5	0.2	0.72	590.0	817.5
Interstitial bin 5AS1‐0.40‐0.75	103.25	126.6	0.2	0.82	516.3	633.0
Distal bin 5AS3‐0.75‐0.97	64.90	94.3	10.2	0.69	6.4	9.2
*Qfhs.ifa‐5A* interval on 5AS	Unknown	350.3	0.9	NA	NA	389.2
5AS chromosome	295.00	384.4	10.6	0.77	27.8	36.3

a Paux *et al*. (2008).

b RSH‐consensus map.

c NI‐RIL map.

### Comparison between meiotic and physical maps

Marker order determined either by recombination events (DH, NI‐RIL) or by induced deletions (RS‐NIL3, RH‐CS) was in complete agreement with the marker assignment to physical deletion bins (Table S3.2). Of the used markers, 45, 32 and 25 were allocated to physical deletion bins C‐5AS1‐0.40, 5AS1‐0.40‐0.75 and 5AS3‐0.75‐0.97, respectively. There was a striking difference in map resolution between meiotic and radiation‐induced deletion maps (Table 2). Unlike genetic linkage maps, where markers clustered at a few recombination points (Figure 1a, b), DSBs in RSH map (Figure 1c) were evenly spaced and separated most of the markers cosegregating in the NI‐RIL and DH maps. Among all markers tested, cfa2250, jfio6 and gpg503 mapped closest to the centromere. The interval between cfa2250 and barc186 in the NI‐RIL map contained seven loci and covered a genetic distance of 0.9 cM. The same interval was separated by 66 loci and covered a distance of 350.3 cR in the RSH‐consensus map. The number of loci increased more than ninefold and estimates for the ratio of cR to cM translate into a 389‐fold increased resolution of the QTL interval for the RSH‐consensus map compared to the meiotic map. A 36.3‐fold improved resolution was calculated for the entire linkage group of 5AS chromosome. Evaluating the physical deletions bins separately resulted in an 817.5‐, 633‐ and 9.2‐fold map improvement for the deletion bins C‐5AS1‐0.40, 5AS1‐0.40‐0.75 and 5AS3‐0.75‐0.97, respectively.

**Figure 4:**
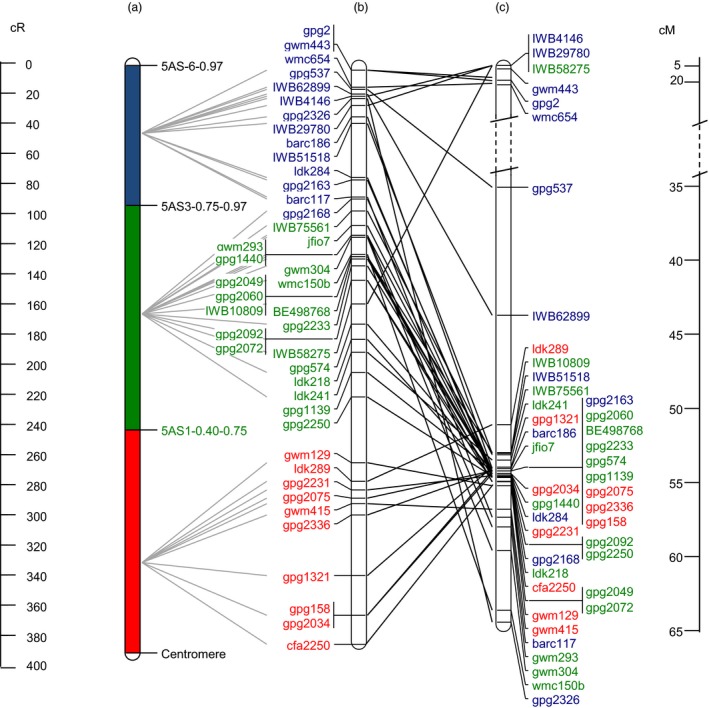
Comparison of markers order and relative distances reported in (a) physical deletion bin map, (b) RSH‐consensus and (c) 5A neighbour map (Barabaschi *et al*., 2015). Only markers analysed across all three maps are included. Solid grey lines connect physical deletion bins to the RSH‐consensus map, and solid black lines connect RSH‐consensus map to the 5A neighbour map. Colours of marker names indicate their physical assignment to deletion bin (red = proximal bin, green = interstitial bin, blue = distal bin). Distances are given on the left (cR) and right (cM).

Forty‐three markers of the RSH‐consensus map are included in the 5AS neighbour map (Barabaschi *et al*., 2015) as well. We compared marker order and relative distance between RSH‐consensus map and the 5AS neighbour map. Considering the neighbour map of the chromosome 5AS, 35 of the 43 markers clustered within a 15 cM distance near the centromere and were tightly linked despite being physically assigned to distal, central and centromeric bins (Figure 4). Physical mapping obtained with the RSH‐consensus map demonstrates a substantial improvement compared to the genetic neighbour map in terms of resolution in marker position. A high rank‐order (ρ = 0.99) but moderate linear relationship (*r *=* *0.50) was calculated between RSH‐consensus and NI‐RIL map (Figure S2a), while only moderate correlation for both rank‐order (ρ = 0.34) and linear relationship (*r *=* *0.40) was obtained between RSH‐consensus and the genetic 5AS neighbour map (Figure S2b). Furthermore, the assignment to genomic scaffolds of TGACv1 whole‐genome assembly has been made for all markers with available sequence (Table S3.3, Data S1). Relating anchored scaffolds of the RSH map with the POPSEQ map (Chapman *et al*., 2015) revealed only for common scaffolds (data not shown). Possibly, this small overlap between maps is due to the fact that we focused on the highly repetitive TE‐rich centromeric interval, while POPSEQ included only the accessible nonrepetitive portion of the genome.

## Discussion

The major FHB resistance QTL *Qfhs.ifa‐5A* and *Qfhi.nau*‐5A (syn *Fhb5*) reside in the pericentromeric interval of chromosome 5A (Buerstmayr *et al*., 2009). Follow‐up studies placed the *Fhb5* to the C‐5AS3‐0.75 bin to a short genetic distance (0.3 cM) while referring to 75% of the physical length of 5AS (Xue *et al*., 2011). To overcome the limitations for high‐resolution fine‐mapping caused by a strongly repressed recombination, we supplemented recombination‐dependent linkage with recombination‐independent RH mapping technique.

### Genetic linkage mapping of 5AS with focus on *Qfhs.ifa‐5A* support interval

Adding more markers to the existing linkage map of chromosome 5A, on the same set of 364 DH lines previously used for *Qfhs.ifa‐5A* mapping (Buerstmayr *et al*. 2002, 2003), failed to obtain new recombination points (Figure S1), while screening the 10‐fold larger NI‐RIL population could separate the markers into smaller subclusters (Figure 1b; Table S3.1). This indicates that, beyond a specific density of markers, map improvement is dependent on numbers of individuals tested. However, even with a very large population, only a limited number of recombinant lines were obtained in the QTL region, producing a few breakpoints only. Among as many as 3650 NI‐RILs, solely four lines showed a recombination within the centromeric C‐5AS1‐0.40 bin, generating a unique recombination point only. This clearly proved fine‐mapping of centromeric intervals via recombination as inapplicable. Recombination around centromeres is highly suppressed and this effect is stronger in chromosomal short arms, where recombination is almost exclusively concentrated in terminal regions and almost absent in proximal and interstitial regions (Akhunov *et al*., 2003; Lukaszewski and Curtis, 1993). Nowadays, high‐throughput techniques are capable to generate abundance of marker data, highlighting the number of recombination events as the most important factor to improve map resolution. For instance, a genetic map developed for chromosome 5A employing the wheat iSelect 90K SNP array (Wang *et al*., 2014) resulted in 91.9% of the 148 markers genetically mapped to 5AS cosegregating and allowing the identification of 12 unique marker loci only (Gadaleta *et al*., 2014). Similarly, although at a much higher marker density, POPSEQ was useful to anchor >40.000 contigs to chromosome 5A that were separated into 61 bins; hence, 99.85% of the contigs remained cosegregating (Chapman *et al*., 2015). Thus, typing more markers will only marginally, if at all, help to refine the position of *Qfhs.ifa‐5A*. For these reasons, in this work genetic fine‐mapping was complemented by radiation hybrid mapping technique.

### Integration of two different radiation‐induced deletion panels to improve the resolution of 5AS pericentromeric region

The radiation selfing panel RS‐NIL3 originated from irradiated seeds of the homozygous line NIL3 containing the *Qfhs.ifa‐5A* resistance allele. This panel was produced not only to improve map resolution, but also to develop viable *in vivo* lines differing for induced deletions across the QTL interval. Irradiated seeds were advanced to the RS_1_ generation to obtain homozygous deletions. In addition to the RS panel, the radiation hybrid panel RH‐CS was used to support high‐resolution mapping, generated by pollinating the aneuploid line CS‐N5AT5B with gamma‐irradiated pollen of the reference cultivar Chinese Spring. An approximately 10‐fold higher proportion of plants had detectable deletions in the RH‐CS panel than in the RS‐NIL3 panel (Table S4); deletions were larger and some of the RH‐CS plants lost the entire chromosome 5A (Figure 3). Several factors in combination may have led to this difference: (i) F_1_ hybrids of the RH‐CS panel are hemizygous for chromosome 5A and thus directly amenable for detecting deletions; instead, RS‐NIL3 plants require at least one selfing generation to obtain deletions in homozygous state; as severe deletions are deleterious and do not survive mitotic and meiotic cell divisions, selfing will purge all lethal and possibly many of the more or less deleterious mutations. The RH‐CS panel was intended for mapping only; therefore, induced deletions need not be transmitted to the next generation, circumventing losses of deletions due to genetic drift and natural selection; (ii) after selfing, on average only one of four plants will be homozygous for the deletion and all heterozygous deletions will remain undetected; (iii) lengths of deleted fragments in the RS‐NIL3 panel were smaller than those detected in the RH‐CS panel and interstitial deletions predominated. While terminal deletions are easily detected by screening the genotypes with markers located at the distal end of the chromosome, interstitial deletions, that are frequently smaller in size, may remain undetected.

The genome of the recipient parent, untreated with γ‐ray, and the three sets of homoeologous subgenomes efficiently buffered radiation‐induced deficiencies of the irradiated pollen source. Abundance of induced deletions using pollen‐irradiated technique has been already reported by Tiwari *et al*. (2012). Pollen irradiation affects cells postmeiosis, minimizing selection against chromosomal aberration in the mature pollen. Thus, using treated pollen as donor for deletions and aneuploid lines as recipient parent appears a very promising technique for high‐resolution mapping.

Sizes of induced deletions are expected to be diverse, ranging from a few bp to several Mb (Morita *et al*., 2009; Naito *et al*., 2005; Tiwari *et al*., 2012). The number and positions of the markers used did not allow for detecting very small deletions; on the other hand, very small deletions produce singletons that do not contribute to marker ordering. As markers in the RS‐NIL3 and RH‐CS maps are ordered via deleted fragments that partially overlap, deletions of varying sizes that encompass several markers are preferred. The differences in position and length of deletions between the two panels perfectly complemented each other, contributing to a more robust map construction. Exploiting data of both panels increased the number of jointly retained/deleted overlapping intervals, facilitated marker ordering and yielded a highly improved map resolution compared to the genetic NI‐RIL map.

Plants of the RS‐NIL3 panel have a common genetic background but differ for induced deletions across the FHB resistance interval of *Qfhs.ifa‐5A*. So far, 80 RS‐NIL3 plants have been identified that contain deletions at the target interval and will be phenotypically evaluated in the next field seasons. This will allow a more precise identification of the *Qfhs.ifa‐5A* QTL interval that currently refers to ≈75% of the short arm (250 Mb), which does not yet permit narrowing down to a manageable number of candidate genes. FHB is a quantitative trait highly influenced by the genetic background (Salameh *et al*., 2011), thus some of the off‐site mutations may interfere with the FHB phenotyping. A low background mutation rate expected in the RS‐NIL3 panel, due to low deletion rate, is most beneficial for phenotyping. RS panels offer a cheap, fast and straightforward tool for selecting deletion lines. They have the great advantage to be generated by selfing, and thus—in contrast to RH panels—being unaffected by the genome of an aneuploid line, such as a nullisomic/tetrasomic line for the chromosome under investigation.

### Marker systems applied

We employed different marker types with a high proportion of ISBP and SSR markers. The high number of these two kinds of markers led to a homogeneous map resolution along the entire linkage group (Table 2). ISBPs and RJMs are based on unique and genome‐specific insertion junctions generated by transposable elements that contribute to approximately 80% of the wheat genome (Dvořák, 2009). TE junctions are randomly distributed along chromosomes, present in both hetero‐ and euchromatin and thus suitable for high‐density RH mapping of the entire genome including centromeres and pericentromeric heterochromatin‐rich regions (Luce *et al*., 2006). Most notably, the highest map resolution was previously obtained in centromere near bins employing RH mapping technique in combination with ISBP markers (Kobayashi *et al*., 2015). In the present work, ISBP, RJM and SSR marker types were evenly distributed along the 5AS chromosome, while no SNP markers were mapped in proximal regions (Table S3.2). A particularly low density of SNPs in the centromeric bin as well as a uniform distribution of SSR markers throughout the map was already reported (Gadaleta *et al*., 2014). Analogously, an underrepresentation of markers in pericentromeric regions was reported employing high‐throughput techniques such as iSelect 90K SNP array or Diversity Array Technology (DArT) markers, leading to the consideration that the unequal distribution of markers and low marker coverage of pericentromeric intervals of the iSelect genotyping platform limited the full mapping potential on whole‐genome radiation hybrid panel (Balcárková *et al*., 2017; Tiwari *et al*., 2012, 2016). SNPs of the iSelect 90K chip, being developed from RNAseq data, are gene‐associated (Wang *et al*., 2014) and DArT markers preferentially target gene‐rich regions (Tiwari *et al*., 2012; Wenzl *et al*., 2004); consequently, the low gene density in centromeric regions (Akhunov *et al*., 2003; Erayman *et al*., 2004; Gill *et al*., 1996) is in agreement with the low marker coverage achieved using these genotyping tools.

### Map comparison

An accurate analysis of the *Qfhs.ifa‐5A* support interval was undertaken through a comparison of all the maps constructed in this work: two genetic maps (DH and NI‐RIL), a physical deletion bin map and three radiation‐induced deletion maps (RS‐NIL3, RH‐CS and thereof derived RSH‐consensus map) (Figure 1). The most limiting factor for genetic linkage mapping was, besides the small number of polymorphic markers, the suppressed recombination at ~70% in the proximal region of chromosome 5A short arm. By contrast, radiation‐induced deletion mapping was able to unequivocally resolve order for most markers. Gamma radiation affects the entire genome causing homogeneous marker loss regardless of positions within the chromosomes (Bassi *et al*., 2013; Riera‐Lizarazu *et al*., 2010; Tiwari *et al*., 2016). Accordingly, radiation‐induced breaks were found to be evenly distributed across the 5AS chromosome, agreeing with previous RH mapping results (Balcárková *et al*., 2017; Bassi *et al*., 2016, 2013; Kalavacharla *et al*., 2006; Kumar *et al*., 2012; 2105; Mazaheri *et al*., 2015; Tiwari *et al*., 2016, 2012). We calculated a 389‐fold increased map resolution for the low‐recombinogenic *Qfhs.ifa‐5A* interval compared to the genetic map and an 817.5‐ and 633‐fold for the centromeric and interstitial bin, respectively. This improvement in resolution was much higher than the 260‐fold (Mazaheri *et al*., 2015) and 136‐fold (Kumar *et al*., 2012) increase previously obtained around wheat centromeres. Moreover, genetic distances were found to provide poor estimates for actual physical distances, while cR distances were much better predictors (Boehnke *et al*., 1991; Newell *et al*., 1998). Accordingly, the repressed recombination rate and the low number of polymorphic markers in centromeric regions caused a strong disproportion between cM and actual physical distances (Table 2). Markers order was in good agreement among NI‐RIL map, deletion bin map and radiation maps; by contrast, discrepancies in the marker order were observed when comparing mapping results with the recently published 5A neighbour map (Figure 4) generated to support anchoring of fingerprinting contigs of the chromosome 5A high‐quality physical map (Barabaschi *et al*., 2015). The discrepancies are probably due to the fact that the neighbour map was constructed combining mapping results of ten different genetic maps and this approach was most likely not sufficient to put in agreement with the genetic order and physical bin location of markers in proximal regions. On the other hand, RSH‐consensus map was able to successfully separate and order markers, despite being genetically tightly or completely linked in the 5A neighbour map, thus much better reflecting their actual physical position on the chromosome.

## Conclusion

Constructing RH panels for anchoring and ordering of contigs for whole‐genome sequence assembling has proved to be an efficient complementary approach, although it may become obsolete when a high‐quality complete wheat genome sequence assembly is available in the near future. Still, high‐resolution and high‐quality RH maps will be among the most valuable tools for identifying discrepancies, and thus validating the genome sequence, especially in the more challenging pericentromeric regions.

Results of the present work clearly demonstrated that, unlike recombination‐dependent genetic linkage mapping, radiation‐induced deletion mapping greatly facilitated mapping of regions with suppressed recombination. Utilization of a radiation selfing panel in combination with a suitable number of markers, here described for the first time, revealed to be particularly suitable for fine‐mapping of recombination‐poor stretches. The high level of plasticity of the wheat genome against chromosomal aberration allowed to generate and select a panel of viable and genetically stable sister lines differing in randomly deleted sequences across the *Qfhs.ifa‐5A* support interval. These lines represent very powerful tools to associate losses of DNA sequences with the FHB phenotype and *vice versa* during evaluation in field test, thus providing an unprecedented tool for fine localization of the *Fhb5* gene locus.

## Experimental procedures

### Plant material and population development

#### Recombination‐based genetic populations

##### Double haploid population (DH)

The same set of 364 recombinant F_1_‐derived DH lines described in Buerstmayr *et al*. (2002, 2003) was subjected to more detailed genotyping across the *Qfhs.ifa‐5A* interval. DH lines descend from a cross between Remus x CM‐82036. Remus (Sappo/Mex//Famos), a German spring wheat cultivar, is highly susceptible to FHB and CM‐82036 (Sumai#3/Thornbird‐S) is highly resistant to FHB and contains the *Qfhs.ifa‐5A* resistance allele.

##### Near‐isogenic recombinant inbred line population (NI‐RIL)

Based on genotypic and phenotypic results, one FHB‐resistant DH line was five times backcrossed to Remus to generate NILs as described in Schweiger *et al*. (2013). The NI‐RIL population was developed by crossing NIL1 to NIL2, having the same genetic background but differing for resistant/susceptible allele at the *Qfhs.ifa‐5A* locus, respectively, allowing the development of 3650 F_2_ plants that were screened for recombinants (as described below).

#### Radiation‐induced deletion panels

##### Radiation selfing panel of NIL3 (RS‐NIL3)

Seeds of the NIL3, carrying the resistance allele for *Qfhs.ifa‐5A*, were irradiated with γ rays at a dosage of 250 Gray (Gy) at the Department of Plant Sciences, North Dakota State University, Fargo, USA. On average, two plants per irradiated seed were propagated to the M_3_ (RS_2_) generation. Additional seeds of NIL3 were irradiated at dosages 240, 270, 300, 330 and 350 Gy at the IAEA (International Atomic Energy Agency) laboratories at Seibersdorf, Austria. The M_1_ (RS_0_) seeds thus obtained were selfed to develop M_2_ (RS_1_) plants. In total, 5157 plants were genotyped composed of 800 M_3_ plants derived from 504 M_1_ seeds irradiated at 250 Gy and 383, 115, 1528, 1195 and 1136 M_2_ plants derived from 383, 115, 325, 324 and 344 M_1_ seeds irradiated at 240, 270, 300, 330 and 350 Gy, respectively (Table S4, Figure S3).

##### Radiation hybrid panel of Chinese Spring (RH‐CS)

Pollen of CS was harvested at flowering and treated with γ‐rays at a dosage of 100 Gy to induce multiple chromosomal deletions. This pollen, which constitutes the RH_0_ generation, was then used to pollinate CS‐N5AT5B (an aneuploid line where both 5A chromosomes are replaced with an additional pair of 5B chromosomes). The resulting RH_1_ generation is hemizygous for 5A, which originates from the irradiated cultivar (Figure S3). Pollen irradiation and crossing were performed at the Department of Plant Sciences, North Dakota State University, Fargo, in collaboration with CREA‐GB, Italy. Seeds were germinated at the IFA‐Tulln laboratory, and 276 RH plants were genotyped with 5A‐specific markers as described below.

### Marker selection

Markers of the short arm of chromosome 5A and localized in the pericentromeric region were specifically selected according to information from published maps (Barabaschi *et al*., 2015; Gadaleta *et al*., 2012, 2014; Somers *et al*., 2004; Wang *et al*., 2014). Sequence information was obtained from published data (Barabaschi *et al*., 2015; Ramirez‐Gonzalez *et al*., 2015) and public databases (http://wheat.pw.usda.gov/GG2/index.shtml). All chosen markers are listed in Table S1. Before screening the populations, markers were tested for their location on chromosome 5A on the following lines: CS‐N5AT5B (Sears, 1966), CS ditelosomic line CS‐DT5AL (Sears and Sears, 1978) and cytogenetic CS deletion lines C‐5AS1‐0.40, 5AS3‐0.75 and 5AS6‐0.97 (Endo and Gill, 1996). The CS‐N5AT5B allowed allocating markers to the 5A chromosome, while CS‐DT5AL, missing the short arm of 5A, was used to reveal the arm location of the markers. Deletion lines C‐5AS1‐0.40, 5AS3‐0.75 and 5AS6‐0.97 having terminal deletions with breakpoints located 40%, 75% and 97% distal to the centromere were used to allocate markers to the corresponding deletion bins. In addition, NIL1, NIL2, NIL3 and CS were included into the screening to detect polymorphic markers localized in the *Qfhs.ifa‐5A* region. PCR products of NIL3 and CS were used as references for fragment sizes of the 5AS alleles when screening RS‐NIL3 and RH‐CS panel.

### Molecular analysis

Total genomic DNA was isolated as previously described (Saghai‐Maroof *et al*., 1984). PCR protocols are described in Data S2.

#### Genotyping of the DH population

The DH‐based linkage map of the 5A group reported in Buerstmayr *et al*. (2003) was supplemented with genotypic data of 13 additional 5A‐specific markers. The linkage map was re‐calculated and QTL re‐estimated based on the refined linkage group using composite interval mapping of the Q‐gene program (version 4.3.10) (Nelson, 1997). For QTL analysis, the same phenotypic data were used as in Buerstmayr *et al*. (2003).

#### Genotyping of the NI‐RIL population

A total of 3650 F_2_ plants were screened for recombinations in the *Qfhs.ifa‐5A* interval using barc186 and barc56 on 5AS and barc1, barc180 and wmc805 on 5AL as flanking markers. Plants containing a recombination were selfed and one homozygous recombinant F_3_ plant per recombinant F_2_ plant was selected for fine‐mapping.

#### Genotyping the RS‐NIL3 and RH‐CS panels

The survey of deletions in RS‐NIL3 and RH‐CS panel was initially performed using 15 and 35 markers, respectively. Plants having at least one 5AS‐specific marker deleted were selected and further analysed using 102 markers located on 5AS and three markers (wmc705, barc1 and gwm595) located on 5AL. Markers located on the long arm were included to determine whether deletions span across the centromere. Wmc705 and barc1 physically map on the central bin 5AL5‐0.46‐0.55, while gwm595 is located on the terminal bin of 5AL, 5AL7‐0.87‐1.00 (Barabaschi *et al*., 2015). Lines having all markers deleted were assumed to lack the entire 5A chromosome. Mandatory for correct scoring of deletions is to distinguish missing amplification due to a chromosomal deletion from PCR failure. Therefore, a multiplex PCR protocol was chosen that simultaneously amplified 5AS‐specific and 5AS‐unspecific sequences in a single PCR. Information on number, sizes and presence of 5A‐specific and 5A‐unspecific amplicons of individual markers was obtained while testing markers, as described in marker selection. Based on this prior knowledge, primer pairs amplifying only 5AS‐specific sequences were combined with primer pairs amplifying both 5AS‐specific and 5A‐unspecific sequences. The 5AS‐unspecific amplicons served as a positive control for a successful PCR. Amplicon‐specific size markers were loaded next to the PCR samples in order to allow for assigning the amplified PCR fragments to the respective markers when separating fragments of the multiplex PCRs (Figure S4). Reactions with missing values or ambiguous results were repeated until clear scores were obtained for data points crucial for assigning a correct marker ordering, for example missing values at the beginning or end of a deletion.

### Map construction

Genetic maps of DH, NI‐RIL, RS‐NIL3 and RH‐CS populations/panels were constructed using CarthaGène 1.2‐LKH (de Givry *et al*., 2005). Maps of RS‐NIL3 and RH‐CS panel were initially calculated using the algorithm for a haploid model, as haploid data sets allow faster ordering of the marker data (Lange *et al*., 1995). The RS‐NIL3 and RH‐CS data sets were then merged by the command *dsmergen* assuming that the data represent a single panel. As markers had already been physically assigned to chromosome 5AS, all markers were taken together to form a single linkage group. Markers showing identical deletion/retention patterns (double markers) were merged to single markers. First marker ordering was derived using 2‐points log‐likelihoods by running the *lkh* commands of the CarthaGène program. This converted the given marker data into a Traveling Salesman Problem using the Lin–Kernighan heuristic (Lin and Kernighan, 1973). Commands *polish* and *flips* were run to find a map whose log‐likelihood is improved over this initial map. The *polish* algorithm removes one marker of the initial map and tries to insert it in all possible intervals. For the *flips* command, a sliding window of five markers was chosen to improve the map by iteratively testing all possible marker orders within this window size. In a second step, the RS‐NIL3, the RH‐CS and the NI‐RIL and DH data sets were merged by the command *dsmergor* producing a consensus marker order, but separate parameter estimates with per‐data set distances. The marker order of the previously obtained best map served as initial marker order for the combined analysis of all four data sets. *Polish* and *flips* command were applied on the complete marker information of all data sets. The Kosambi mapping function was used for calculating centiMorgan (cM) distances between markers in the DH and the NI‐RIL map. Finally, marker distances given in centiRay (cR) of RS‐NIL3 and RH‐CS were determined using the algorithm for the diploid model for both panels separately (*dsmergor*) and for the RSH‐consensus panel (*dsmergen*) assuming that plants of RS‐NIL3 and RH‐CS are members of the same panel. Mapping distance of 1 cR equals a 1% frequency of a breakage occurring between two markers after exposure to a specific radiation dose (Hukriede *et al*., 1999).

### Characterizing the RS‐NIL3 and RH‐CS panels

RH panels were characterized for retention frequency and sizes of deletions. Retention frequency is defined as the proportion of markers retained among all plants tested and was estimated individually for each marker and across panels. Only informative lines (lines having at least one but not all markers deleted) were used for calculating the retention frequencies. Lines having all markers deleted (whole chromosome 5A missing) or no markers deleted were excluded from further analyses. Maximum and minimum sizes (using cR distances of the RSH‐consensus map) of the deleted fragments were calculated as the distance between the position of retained markers (maximum distance) and deleted markers (minimum distance) flanking the deletions. Average lengths of deletions were calculated as the mean of maximum and minimum distances. Map resolution was determined as the ratio of Mb to cR and improvement in map resolution as the ratio of cR to cM.

### Map comparison

Generated maps were compared among each other and to the 5AS physical deletion bin map (Endo and Gill, 1996) and the 5A neighbour map (Barabaschi *et al*., 2015). Relationship of the RSH‐consensus map to the NI‐RIL map and to the 5A neighbour map, in terms of markers order and distances, was calculated using Spearman rank‐order and Pearson product–moment correlation coefficients. Linkage groups and map comparison were drawn with MapChart v2.2 (Voorrips, 2002).

All markers with available sequence (Data S1) were BLAST‐searched against the TGACv1 whole‐genome assembly generated by the Earlham Institute, formerly The Centre for Genome Analysis (TGAC). The BLAST searches were conducted in the dedicated platform at EnsemblPlants website (http://plants.ensembl.org/Triticum_aestivum/Tools/Blast?db=core), considering an identity percentage ≥ than 95%. The position of the obtained TGAC scaffolds was searched along POPSEQ data (Chapman *et al*., 2015).

## Supporting information


**Figure S1** Refined linkage group of the DH population and LOD curves of Fusarium head blight resistance traits.

**Figure S2** Scatterplots of marker positions of the RHS‐consensus map against the NI‐RIL and the 5A neighbour map.

**Figure S3** Development of radiation selfing panel (RS‐NIL3) and radiation hybrid panel (RH‐CS).

**Figure S4** Image of multiplex PCR for primer combination ldk243/ldk284 separated on acrylamide gel.

**Table S1** Results of markers tested for polymorphism between Remus and CM‐82036.

**Table S2** Summary of QTL analysis at *Qfhs.if*a*‐5A* obtained with composite interval mapping.

**Table S3** Genotypic data, map comparison, primer sequences and anchored TGCAv1 scaffolds.

**Table S4** Summary of pre‐screening the RS‐NIL3 and RH‐CS panel.

**Data S1** FASTA file of markers sequences.

**Data S2** PCR protocol.
