# Supplementary material for: High‐resolution mapping of the pericentromeric region on wheat chromosome arm 5AS harbouring the Fusarium head blight resistance QTL Qfhs.ifa‐5A
Source: Plant Biotechnol J. 2017 Nov 10;16(5):1046–56. doi: 10.1111/pbi.12850 (PMC5902775; doi:10.1111/pbi.12850)
Supplement: Supplementary file 1 — Figure S1 Refined linkage group of the DH population and LOD curves of Fusarium head blight resistance traits. [file PBI-16-1046-s010.pdf]

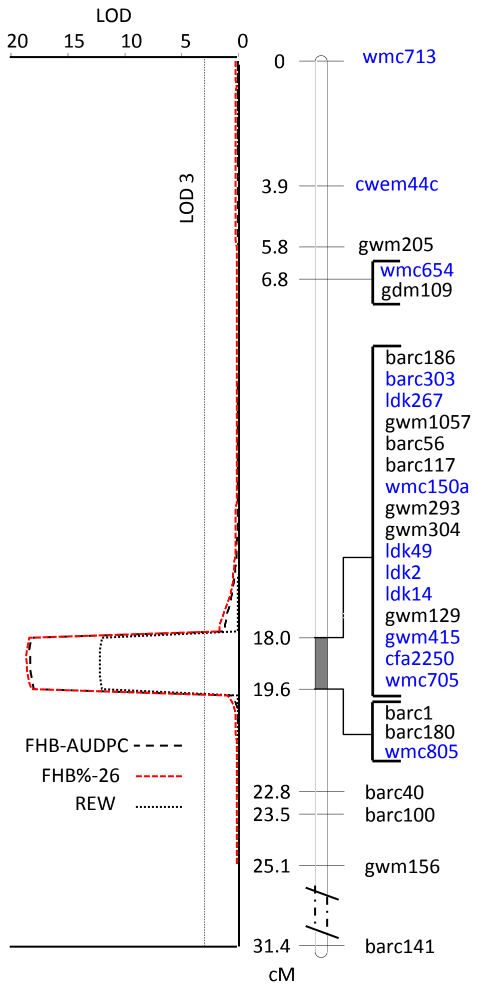

Figure S1 Refined linkage group of the DH population. New added markers are in blue. LOD curves determined by composite interval mapping for FHB area under the disease progress curve (FHB-AUDPC), percentage of infected spikelets on day 26 (FHB%-26), relative ear weight (REW) are shown on the left. Highlighted grey intervals in chromosome bars refer to the *Qfhs.ifa-5A* support interval. For QTL analysis the same phenotypic data were used as in Buerstmayr et al. (2003)
