# Supplementary material for: High‐resolution mapping of the pericentromeric region on wheat chromosome arm 5AS harbouring the Fusarium head blight resistance QTL Qfhs.ifa‐5A
Source: Plant Biotechnol J. 2017 Nov 10;16(5):1046–56. doi: 10.1111/pbi.12850 (PMC5902775; doi:10.1111/pbi.12850)
Supplement: Supplementary file 2 — Figure S2 Scatterplots of marker positions of the RHS‐consensus map against the NI‐RIL and the 5A neighbour map. [file PBI-16-1046-s009.pdf]

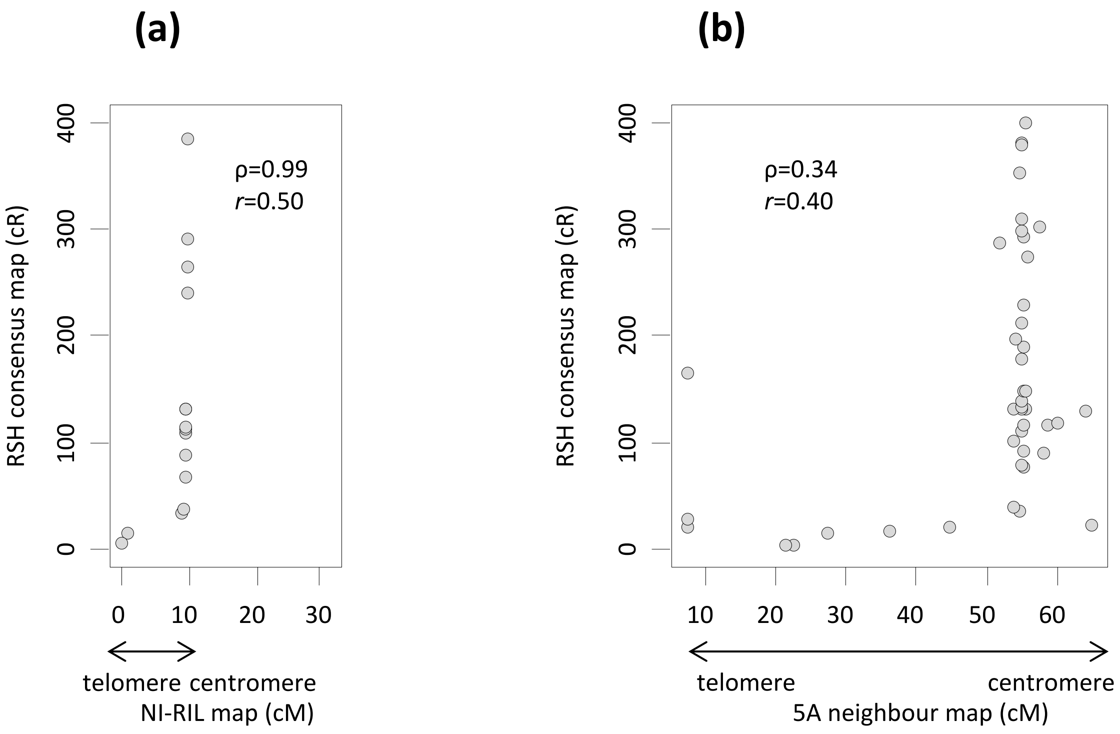

Figure S2 Scatterplots of marker positions of the RHS-consensus map (cR) against (**a**) the NI-RIL map (cM) and against (**b**) the 5A neighbour map (cM) along the chromosome 5AS. Genetic cM distances of 5A neighbour map are derived from Barabaschi et al. (2015)
