# Supplementary material for: High‐resolution mapping of the pericentromeric region on wheat chromosome arm 5AS harbouring the Fusarium head blight resistance QTL Qfhs.ifa‐5A
Source: Plant Biotechnol J. 2017 Nov 10;16(5):1046–56. doi: 10.1111/pbi.12850 (PMC5902775; doi:10.1111/pbi.12850)
Supplement: Supplementary file 3 — Figure S3 Development of radiation selfing panel (RS‐NIL3) and radiation hybrid panel (RH‐CS). [file PBI-16-1046-s008.pdf]

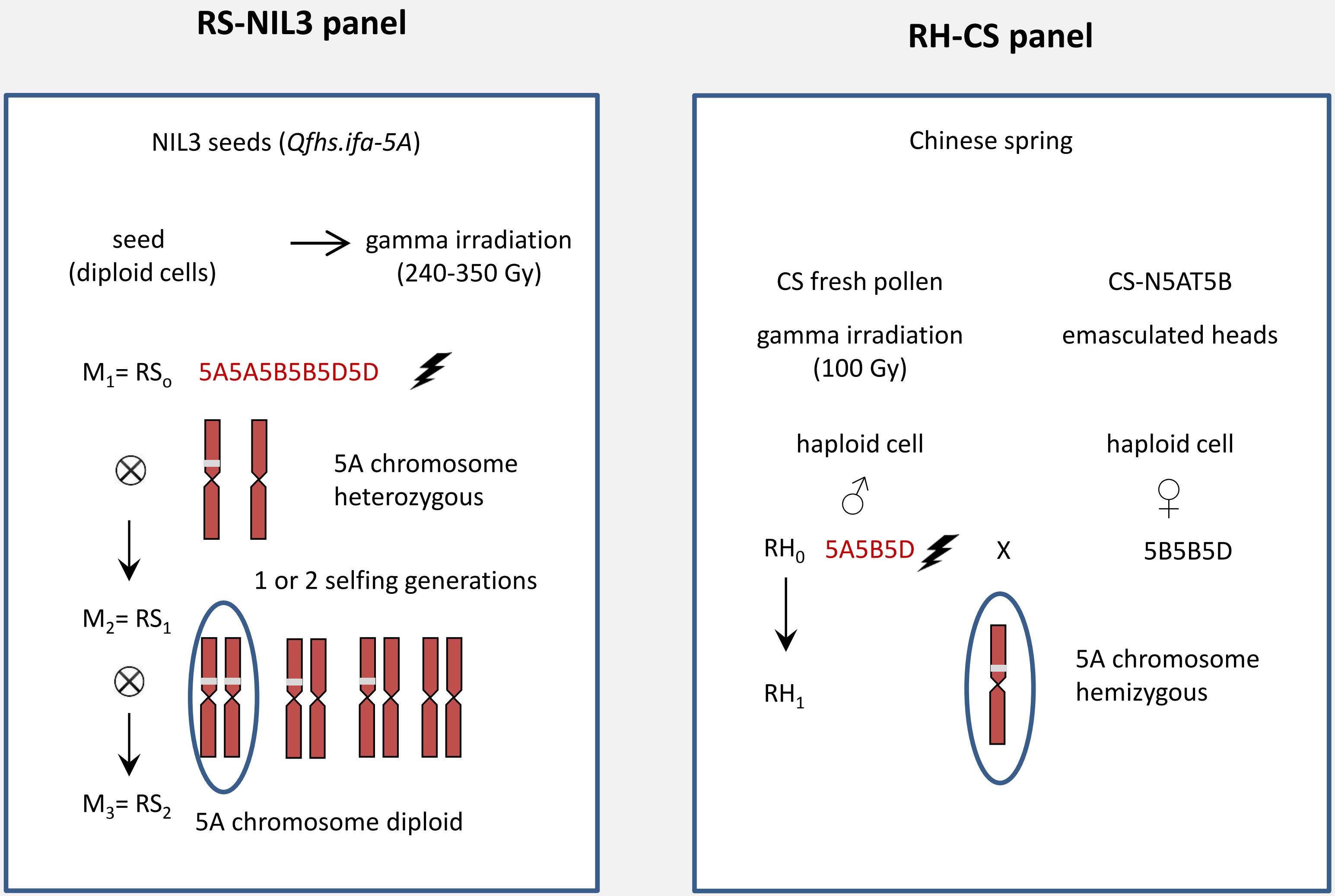

Figure S3 Development of radiation selfing panel (RS-NIL3) and radiation hybrid panel (RH-CS). Development of RS-NIL3 panel involved seed irradiation (M_1 = RS_0) following 1 or 2 generations of selfing (M_2 = RS_1, M_3 = RS_2) to obtain homozygote deletions. RH-CS panel was developed by pollinating Chinese Spring Nulli5ATetra5B (CS-N5AT5B) with irradiated pollen from Chinese Spring (RH_0) to obtain RH_1 plants. RH_1 plants have the 5A chromosome in hemizygous state and potential deletions on chromosome 5A are directly detectable.
