# Supplementary material for: High‐resolution mapping of the pericentromeric region on wheat chromosome arm 5AS harbouring the Fusarium head blight resistance QTL Qfhs.ifa‐5A
Source: Plant Biotechnol J. 2017 Nov 10;16(5):1046–56. doi: 10.1111/pbi.12850 (PMC5902775; doi:10.1111/pbi.12850)
Supplement: Supplementary file 6 — Table S2 Summary of QTL analysis at Qfhs.ifa‐5A obtained with composite interval mapping. [file PBI-16-1046-s005.docx]

| **Table S2** Summary of QTL analysis at *Qfhs.if*a*-5A* obtained with composite interval mapping. QTL estimates for mean values of percentage of infected spikelets on day 26 after inoculation (FHB%-26), the area under the disease progress curve (AUDPC) for individual years and across experiments in Tulln and the relative ear weight (REW) over 2 years of the experiments in Tulln, and for the FHB%-mean in Hohenheim in 2001. For details see Buerstmayr et al. (2003) | | | | | | | | |
| --- | --- | --- | --- | --- | --- | --- | --- | --- |
| trait | | experiment |  | flanking markers | |  | LOD | VE%^2^ |
| FHB%-26 | | | | | | | | |
|  |  | overall mean^1^ |  | barc186 | wmc805 |  | 18.6 | 0.28 |
|  |  | mean 1999 |  | barc186 | wmc805 |  | 21.2 | 0.31 |
|  |  | mean 2001 |  | barc186 | wmc805 |  | 8.0 | 0.13 |
| AUDPC | | | | | | | | |
|  |  | overall mean^1^ |  | barc186 | wmc805 |  | 18.6 | 0.28 |
|  |  | mean 1999 |  | barc186 | wmc805 |  | 21.5 | 0.31 |
|  |  | mean 2001 |  | barc186 | wmc805 |  | 8.7 | 0.14 |
| REW | | | | | | | | |
|  |  | overall mean^1^ |  | barc186 | wmc805 |  | 12.2 | 0.19 |
| FHB%-mean | | | | | | | | |
|  |  | Hohenheim 2001 |  | barc186 | wmc805 |  | 8.10 | 0.13 |
| ^1^ Tulln (means over 1999 and 2001) | | | |  |  |  |  |  |
| ^2^ percentage of explained phenotypic variance | | | | |  |  |  |  |
