# Supplementary material for: High‐resolution mapping of the pericentromeric region on wheat chromosome arm 5AS harbouring the Fusarium head blight resistance QTL Qfhs.ifa‐5A
Source: Plant Biotechnol J. 2017 Nov 10;16(5):1046–56. doi: 10.1111/pbi.12850 (PMC5902775; doi:10.1111/pbi.12850)
Supplement: Supplementary file 8 — Table S4 Summary of pre‐screening the RS‐NIL3 and RH‐CS panel. [file PBI-16-1046-s002.docx]

**Table S4** Identified plants containing deletions after pre-screening of all genotypes of RS-NIL3 and RH-CS panel with 15 and 35 markers, respectively

|  |  |  |  | number of genotypes analysed |  | genotypes with ≥ 1 marker deleted | | |
| --- | --- | --- | --- | --- | --- | --- | --- | --- |
|  |  |  |  |  |  | number |  | % |
| **RS-NIL3 panel ─ seed irradiated** | | | | |  |  |  |  |
|  | generation | dosage (Gy) |  |  |  |  |  |  |
|  | RS_2_(M_3_) | 250 |  | 800 |  | 19 |  | 2.38 |
|  | RS_1_(M_2_) | 240 |  | 383 |  | 4 |  | 1.04 |
|  | RS_1_(M_2_) | 270 |  | 115 |  | 2 |  | 1.74 |
|  | RS_1_(M_2_) | 300 |  | 1528 |  | 25 |  | 1.64 |
|  | RS_1_(M_2_) | 330 |  | 1195 |  | 20 |  | 1.67 |
|  | RS_1_(M_2_) | 350 |  | 1136 |  | 25 |  | 2.20 |
|  | **Total number** | |  | **5157** |  | **95** |  | **1.84** |
| **RH-CS panel ─ pollen irradiated** | | | | |  |  |  |  |
|  | **RH_1_** | **100** |  | **276** |  | **50** |  | **18.12** |
