# Supplementary material for: High‐resolution mapping of the pericentromeric region on wheat chromosome arm 5AS harbouring the Fusarium head blight resistance QTL Qfhs.ifa‐5A
Source: Plant Biotechnol J. 2017 Nov 10;16(5):1046–56. doi: 10.1111/pbi.12850 (PMC5902775; doi:10.1111/pbi.12850)
Supplement: Supplementary file 4 — Figure S4 Image of multiplex PCR for primer combination ldk243/ldk284 separated on acrylamide gel. [file PBI-16-1046-s007.pdf]

Figure S4 Example of multiplex PCR for primer-combination ldk243/ldk284

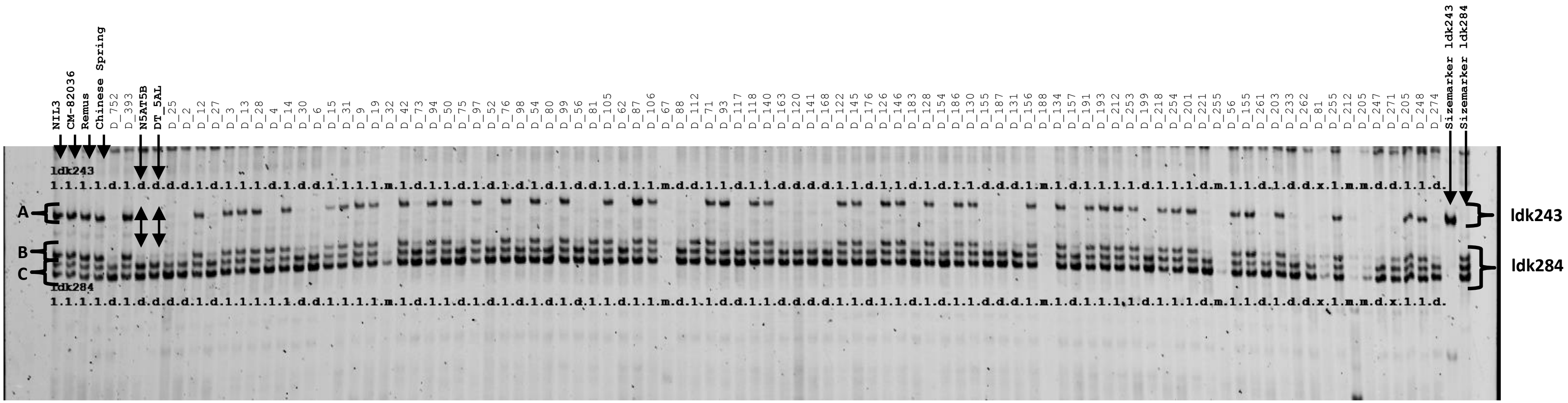

Figure S4 Image of multiplex PCR for primer combination ldk243/ldk284 separated on acrylamidgel. Non irradiated control lines NIL3, CM-82036, Remus, Chinese spring, N5AT5B and DTEL-5AL are on the left, size markers ldk243 and ldk284 on the very right side (indicated by arrows). Ldk243 amplifies a sequence on 5AS (A) and ldk284 amplifies both, a 5AS specific (B) and two non 5AS specific sequences (C). Unspecific fragments of primer ldk284 (C) are used as positive controls for successful PCR reaction. Retained 5AS sequences are scored as 1, deleted as d, PCR failure as m (missing) and unsure scores as x. Size markers had been produced for each marker separately (using DNA from non-irradiated NIL3) prior screening the RS and RH panel.
