# Supplementary material for: High‐resolution mapping of the pericentromeric region on wheat chromosome arm 5AS harbouring the Fusarium head blight resistance QTL Qfhs.ifa‐5A
Source: Plant Biotechnol J. 2017 Nov 10;16(5):1046–56. doi: 10.1111/pbi.12850 (PMC5902775; doi:10.1111/pbi.12850)
Supplement: Supplementary file 10 — Data S2 PCR protocol. [file PBI-16-1046-s004.pdf]

Supplemental File S2

PCR protocol

Polymerase chain reactions (PCRs) were performed in a final volume of 10 µl. Reactions contained 30 ng of genomic DNA, 1x PCR buffer, 1.5 mM $MgCl_2$, 200 nM of each dNTP, 2 pmol reverse primer, 0.2 pmol forward primer (5´ end tailed with the M13 primer sequence), 1.8 pmol M13 primer (CCCAGTCACGACGTTG) fluorescence labelled with either FAM or Cy5, and 1.0 unit of Taq DNA polymerase. Reaction mix of multiplex PCR was essentially the same, with 2 pmol reverse and 0.2 pmol forward primer of each locus specific marker and 3 pmol fluorescence labelled M13 primer. The PCR reaction cocktails were prepared and kept on ice and placed into pre heated cycler (95 °C) immediately after aliquoting PCR master mix into wells containing DNA to avoid amplification of non-specific DNA sequences (manual hot-start PCR).

The touchdown PCR program had an initial denaturation step at 95°C for 5 min, followed by seven cycles of 95°C for 50 seconds, 65°C for 60 seconds (reduced by 2°C per cycle), 72°C for 60 seconds, 25 cycles of 95°C for 30 seconds, 51°C for 30 seconds, 72°C for 30 seconds with a final extension at 72 °C for 5 min.

PCR amplicons were separated on non-denaturing 12% polyacrylamide gels prepared with 1 x TBE as the gel buffer in a vertical C.B.S. electrophoresis chamber (C.B.S. Scientific Inc., Del Mar, CA, USA). Gels were run under constant 400 V electrophoresis conditions at 10°C for 2 h.

Gels were scanned on a Typhoon-TRIO fluorescence scanner (GE Healthcare, http://www.gehealthcare.com) in fluorescent mode.
